# Clinical Natural Language Processing in languages other than English: opportunities and challenges

**DOI:** 10.1186/s13326-018-0179-8

**Published:** 2018-03-30

**Authors:** Aurélie Névéol, Hercules Dalianis, Sumithra Velupillai, Guergana Savova, Pierre Zweigenbaum

**Affiliations:** 1 LIMSI, CNRS, Université Paris Saclay, Rue John von Neumann, Paris, F-91405 Orsay France; 2 DSV, Stockholm University, Kista, Sweden; 3 School of Computer Science and Communication, KTH, Stockholm, Sweden; 4 Institute of Psychiatry, Psychology and Neuroscience, King’s College, London, UK; 5 Children’s Hospital Boston and Harvard Medical School, Boston, Massachusetts, USA

**Keywords:** Natural Language Processing, Clinical Decision-Making, Languages other than English

## Abstract

**Background:**

Natural language processing applied to clinical text or aimed at a clinical outcome has been thriving in recent years. This paper offers the first broad overview of clinical Natural Language Processing (NLP) for languages other than English. Recent studies are summarized to offer insights and outline opportunities in this area.

**Main Body:**

We envision three groups of intended readers: (1) NLP researchers leveraging experience gained in other languages, (2) NLP researchers faced with establishing clinical text processing in a language other than English, and (3) clinical informatics researchers and practitioners looking for resources in their languages in order to apply NLP techniques and tools to clinical practice and/or investigation. We review work in clinical NLP in languages other than English. We classify these studies into three groups: (i) studies describing the development of new NLP systems or components de novo, (ii) studies describing the adaptation of NLP architectures developed for English to another language, and (iii) studies focusing on a particular clinical application.

**Conclusion:**

We show the advantages and drawbacks of each method, and highlight the appropriate application context. Finally, we identify major challenges and opportunities that will affect the impact of NLP on clinical practice and public health studies in a context that encompasses English as well as other languages.

## Background

### Clinical research in a global context

Healthcare is a top priority for every country. The goal of clinical research is to address diseases with efforts matching the relative burden [1]. Computational methods enable clinical research and have shown great success in advancing clinical research in areas such as drug repositioning [2]. Much clinical information is currently contained in the free text of scientific publications and clinical records. For this reason, Natural Language Processing (NLP) has been increasingly impacting biomedical research [3–5]. Prime clinical applications for NLP include assisting healthcare professionals with retrospective studies and clinical decision making [6, 7]. There have been a number of success stories in various biomedical NLP applications in English [8–19]. The ability to analyze clinical text in languages other than English opens access to important medical data concerning cohorts of patients who are treated in countries where English is not the official language, or in generating global cohorts especially for rare diseases. One such example is the Phelan-McDermid Syndrome Foundation (PMSF), which is leading a Patient Powered Research Network project (part of the Patient Centered Outcome Research Institute, PCORI [20] on a very rare disease. PMSF parents, together with researchers and advisors, launched an international patient registry, the PMSIR, that is directed, governed, and implemented by patient families. There are a total of 900 cases of this rare disease in the entire world. Each patient contributed their EHR and genomics data to enable phenotype/genotype studies. Recently, Kohane et al. have shown that methods allowing an aggregated exploitation of clinical data from multiple healthcare centers could contribute to make headway in the understanding of autism spectrum disorders [21]. Cross-lingual text mining of newswires in thirteen languages was shown to be helpful for automated health surveillance of disease outbreaks, and was routinely implemented in the BioCaster portal [22].

In this context, data extracted from clinical text and clinically relevant texts in languages other than English adds another dimension to data aggregation. The World Health Organization (WHO) is taking advantage of this opportunity with the development of IRIS [23], a free software tool for interactively coding causes of death from clinical documents in seven languages. The system comprises language-dependent modules for processing death certificates in each of the supported languages. The result of language processing is standardized coding of causes of death in the form of ICD10 codes, independent of the languages and countries of origin.

### Objective and Scope

This paper follows-up on a panel discussion at the 2014 American Medical Informatics Association (AMIA) Fall Symposium [24]. Following the definition of the International Medical Informatics Association (IMIA) Yearbook [25, 26], clinical NLP is a sub-field of NLP applied to clinical texts or aimed at a clinical outcome. This encompasses NLP applied to texts in Electronic Health Records (EHRs), but also extends to the development of resources for clinical NLP systems, and to clinically relevant research addressing biomedical information retrieval or the analysis of patient-authored text for public health or diagnostic purposes. We survey studies conducted over the past decade and seek to provide insight on the major developments in the clinical NLP field for languages other than English. We outline efforts describing (i) building new NLP systems or components from scratch, (ii) adapting NLP architectures developed for English to another language, and (iii) applying NLP approaches to clinical use cases in a language other than English.

Finally, we identify major NLP challenges and opportunities with impact on clinical practice and public health studies accounting for language diversity.

## Main Text

### Review method and selection criteria

Conducting a comprehensive survey of clinical NLP work for languages other than English is not a straightforward task because relevant studies are scattered across the literature of multiple fields, including medical informatics, NLP and computer science. In addition, the language addressed in these studies is not always listed in the title or abstract of articles, making it difficult to build search queries with high sensitivity and specificity.

In order to approximate the publication trends in the field, we used very broad queries. A Pubmed query for “Natural Language Processing” returns 4,486 results (as of January 13, 2017). Table 1 shows an overview of clinical NLP publications on languages other than English, which amount to almost 10% of the total.

**Table 1:** Number of publications returned by a PubMed search for “Natural Language Processing AND *language* [tiab]” where *language* is instantiated with a specific language name, on January 13, 2017 along with references cited in this review for each language. The last row (bolded) presents overall information for all languages studied in this review

Language (ISO 639-1 language code)	PubMed Count	Cited in this review
French (FR)	111	[31, 77]*[71, 160, 161]*[158]*[65]*[78]*[66, 79, 94]
		[156]* [50, 67, 89, 109, 120]* [54]* [154]* [159]*
		[140] [138]* [7, 56, 59, 60, 90, 116, 117] [163]*
		[112] [70]* [126, 152, 153] [55]*
German (DE)	69	[31]*[115] [72]*[156]* [154]*[141] [109]*[118] [138]*
		[84] [163]* [27, 53, 88] [70]* [80, 124] [36, 106]
Chinese (ZH)	54	[155]* [68, 73, 96] [154]* [42, 43, 69, 99] [103, 122]
Spanish (ES)	39	[161]*[158]*[155]*[156]*[54]*[154]*[138]
		*[30, 98, 107, 119] [70]*[58] [34, 108], [55, 157]*
Japanese (JA)	30	[158]*[33, 37] [154]* [49, 127, 149, 151],
Dutch (DU)	20	[114] [139] [138]*[110] [70]*
Swedish (SV)	15	[57, 104] [74]*[92] [109]* [48, 61, 105]
		[35, 93, 113, 123]
Portuguese (PT)	14	[28, 83], [55]*
Greek (EL)	14	[52]
Italian (IT)	12	[46, 47, 97]
Korean (KO)	11	[155]*[91]
Arabic (AR)	9	[158]*[162]
Finnish (FI)	9	[38, 40] [74]*[32, 85, 121]
Czech (CS), Russian (RU)	7	[155]*, [163]*
Polish (PL)	6	[29, 82], [156]*
Hebrew (HE)	5	[41, 44]
Danish (DA)	4	[86, 87] [45]
Turkish (TR)	3	[156]*
Bulgarian (BG)	2	[62, 64, 95, 100–102]
Basque (EU)	1	[51]
Georgian (KA)	1	[125]
Hungarian (HU)	0	[156]*
**Overall**	**435**	**114**

Note that some included articles are not indexed in MEDLINE but in other publication venues such as ACL. A star indicates work that addresses several languages

We are showing the results of this query as an imperfect proxy for estimating the scale of the biomedical literature relevant to NLP research, as some publications addressing clinical NLP may not appear in PubMed, and some publications referenced in PubMed may be missed by the query. As described below, our selection of studies reviewed herein extends to articles not retrieved by the query.

**Fig. 1:**
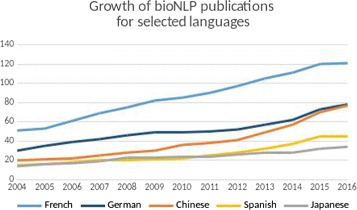
Growth of bio-clinical NLP publications in MEDLINE over the past decade, for the top 5 studied languages other than English

Figure 1 shows the evolution of the number of NLP publications in PubMed for the top five languages other than English over the past decade. We can see that French benefits from a historical but sustained and steady interest. Chinese and Spanish have recently attracted sustained efforts. Japanese and German seem to receive plateauing attention.

This work is not a systematic review of the clinical NLP literature, but rather aims at presenting a selection of studies covering a representative (albeit not exhaustive) number of languages, topics and methods. We browsed the results of broad queries for clinical NLP in MEDLINE and ACL anthology [26], as well as the table of contents of the recent issues of key journals. We also leveraged our own knowledge of the literature in clinical NLP in languages other than English. Finally, we solicited additional references from colleagues currently working in the field.

Our selection criteria were based on the IMIA definition of clinical NLP [25, 26]. For instance, the broad queries employed in MEDLINE resulted in a number of publications reporting work on speech or neurobiology, not on clinical text processing, which we excluded. Moreover, with the increased volume of publications in this area in the last decade, we prioritized the inclusion of studies from the past decade. In total, 114 publications across a wide range of languages fulfilled these criteria (Table 1).

### Clinical NLP in languages other than English

This section reviews the topics covered by recently published research on clinical NLP which addresses languages other than English. We organize the section by the type of strategies used in the specific studies. Table 2 presents a classification of the studies cross-referenced by NLP method and language.

**Table 2:** List of studies presented in this review categorized by NLP method used and language(s) addressed

Method/Task	Language/reference cited in this review
Core NLP	
- Morphology	FR [78] PL [29]
- Part of Speech tagging	PT [28] ES [30]
- Parsing	FI [32, 38, 40] FR [31, 77] GR [52] JA [33, 37]
- Segmentation	DE [27] HE [41]
Resource development	
- Lexicons	BG [62] EL [52] EU [51] FR [50, 65–67] HE [44] JA [49] SV[48] ZH [68, 69]
- Corpora and annotation	EL [52] EN-{FR,ES} [54] EN-{ES,FR,PT} [55]
	ES [58] FR [59, 117]
- Models, methods	DE [53] FR [60]
De-identification	FR [56, 79, 89, 90] KO [91] SV [57, 92]
Information extraction	
- Medical Concepts	BG [62, 64] ZH [42, 43] DE [80, 84, 115, 124] DU [114] ES [34]
	IT [46, 47, 97] PL [82] SV [61]
- Findings/Symptoms	DE [118], SV [61, 93] ZH [96]
- Drugs/Adverse events	BG [95, 102] DA [87] ES [98] FR [94, 116] SV [61]
- Specific characteristics	EN-{ZH,FR,DE,JA,ES} [154] FR [120] ZH [99] DU [114]
- Relations	BG [64] DE [84, 115] IT [46, 47]
Classification	
- Phenotyping from EHR text	BG [100] ES [119] FI [121] FR [7, 126] KA [125] PT [83]
	SV [123] ZH [122]
- Indexing and coding	EN-FR [71] FI [85] FR [153] JA [149, 151]
- Patient-authored text	JA [127]
- Cohort stratification	DA [86] DE [88]
Context Analysis	DU [110]
- Negation detection	BG [101] DA [45] DE [106] DU [110] ES [107, 108]
	FR,DE,SV [109] SV [104, 105]
- Uncertainty/Assertion	SV [105] ZH [103]
- Temporality	FR [112] SV [113]
- Abbreviation	DE [36] SV [35]
- Experiencer	DU [110]
Multilingual tasks	
- Translation	EN-ES [157] EN-FR [159] EN-{KO,RU,ES,ZH} [155]
	EN-{FR,DE,HU,PL,ES,TU} [156], FR-DE [158]
- Information Retrieval	AR [162] FR [160], EN-{CZ,DE,FR} [163] EN-{ES,FR} [161]
- Cultural analysis	DE [72], EN-ZH [73], FI-SV [74]
Shared tasks	
- CLEF-ER2013	DE,DU,FR,ES- [138]
- CLEF eHealth 2015, 2016	FR [152, 153]
- NTCIR 2014, 2016	JA [149, 151]

The two letter language codes are introduced in Table 1. When multiples languages are addressed in one paper we provide a comma separated list; dashes mark language pairs in multilingual work

#### Building new systems and resources

**New NLP systems or components**

Some of the work in languages other than English addresses core NLP tasks that have been widely studied for English, such as sentence boundary detection [27], part of speech tagging [28–30], parsing [31, 32], or sequence segmentation [30]. Word segmentation issues are more obviously visible in languages which do not mark word boundaries with clear separators such as white spaces. This is the case, for instance, in Chinese, Japanese, Vietnamese and Thai. A study of automatic word segmentation in Japanese addressed the lack of spacing between words in this language [33]. The authors implemented a probabilistic model of word segmentation using dictionaries. Abbreviations are common in clinical text in many languages and require term identification and normalization strategies. These have been studied for Spanish [34], Swedish [35], German [27, 36] and Japanese [37]. More complex semantic parsing tasks have been addressed in Finnish [38] through the addition of a PropBank layer [39] to clinical Finnish text parsed by a dependency parser [40].

Core NLP tasks are sometimes evaluated as part of more complex tasks. For instance, a study on Hebrew medical text shows that segmentation methods accounting for transliterated words yield up to 29% performance improvement in medical term extraction [41]. Word segmentation was also shown to outperform character segmentation for named entity recognition in Chinese clinical text. In addition, performing segmentation and named entity recognition jointly yielded a 1% improvement for both. The overall performance of named entity recognition using these special features was above 0.90 F1-measure for four entity types, a performance comparable to English state-of-the-art [42, 43]. Conversely, in an effort addressing the expansion of English abbreviations in Japanese text [37] a study on eight short forms associated to two or more long forms found that character (vs. word) segmentation performed better for the task. However, it can be argued that in the context of code-switching and transliteration (English abbreviations appeared verbatim in Japanese text, accompanied by an expanded form of the acronym in Japanese), the distribution of words and characters made the text sufficiently different from standard Japanese to warrant specific processing. Cohen et al. [44] studied the impact of the high frequency of transliterated terms in Hebrew clinical narratives. They report that the use of a semi-automatically acquired medical dictionary of transliterated terms improves the performance of information extraction. The effect of spelling correction and negation detection on an ICD10 coding system was studied for Danish and both features were found to yield improved performance [45].

**Lexicons, terminologies and annotated corpora** While the lack of language specific resources is sometimes addressed by investigating unsupervised methods [46, 47], many clinical NLP methods rely on language-specific resources. As a result, the creation of resources such as synonym or abbreviation lexicons [27, 36, 48] receives a lot of effort, as it serves as the basis for more advanced NLP and text mining work.

Distributional semantics was used to create a semantic space of Japanese patient blogs, seed terms from the categories Medical Finding, Pharmaceutical Drug and Body Part were used to expand the vocabularies with promising results [49].

There is sustained interest in terminology development and the integration of terminologies and ontologies in the UMLS [50], or SNOMED-CT for languages such as Basque [51]. In other cases, full resource suites including terminologies, NLP modules, and corpora have been developed, such as for Greek [52] and German [53].

The development of reference corpora is also key for both method development and evaluation. Recently, researchers produced annotated corpora for tasks such as machine translation [54, 55], de-identification in French [56] and Swedish [57], drug-drug interaction in Spanish [58], named entity recognition and normalization for French [59], and also for linguistic elements such as verbal propositions and arguments for Finnish [38]. The study of annotation methods and optimal uses of annotated corpora has been growing increasingly with the growth of statistical NLP methods [7, 60, 61].

For some languages, a mixture of Latin and English terminology in addition to the local language is routinely used in clinical practice. This adds a layer of complexity to the task of building resources and exploiting them for downstream applications such as information extraction. For instance, in Bulgarian EHRs medical terminology appears in Cyrillic (Bulgarian terms) and Latin (Latin and English terms). This situation calls for the development of specific resources including corpora annotated for abbreviations and translations of terms in Latin-Bulgarian-English [62]. The use of terminology originating from Latin and Greek can also influence the local language use in clinical text, such as affix patterns [63].

Multilingual corpora are used for terminological resource construction [64] with parallel [65–67] or comparable [68, 69] corpora, as a contribution to bridging the gap between the scope of resources available in English vs. other languages. More generally, parallel corpora also make possible the transfer of annotations from English to other languages, with applications for terminology development as well as clinical named entity recognition and normalization [70]. They can also be used for comparative evaluation of methods in different languages [71].

A notable use of multilingual corpora is the study of clinical, cultural and linguistic differences across countries. A study of forum corpora showed that breast cancer information supplied to patients differs in Germany vs. the United Kingdom [72]. Furthermore, a study of clinical documents in English and Chinese evidenced a lower density of treatment concepts in Chinese documents [73] which was interpreted as a reflection of cultural differences between clinical narrative styles and suggests that this needs to be accounted for when designing clinical NLP systems for Chinese.

Conversely, a comparative study of intensive care nursing notes in Finnish vs. Swedish hospitals showed that there are essentially linguistic differences while the content and style of the documents is similar [74].

**Adapting NLP architectures developed for English**

Studying sublanguages, Harris [75] observed that “The structure of each science language is found to conform to the information in that science rather than to the grammar of the whole language.” Sager’s LSP system [76], developed for the syntactic analysis of medical English, was adapted to French [77]. Deléger et al. [78] also describe how a knowledge-based morphosemantic parser could be ported from French to English.

This shows that adapting systems that work well for English to another language could be a promising path. In practice, it has been carried out with varying levels of success depending on the task, language and system design. The importance of system design was evidenced in a study attempting to adapt a rule-based de-identification method for clinical narratives in English to French [79]. Language-specific rules were encoded together with de-identification rules. As a result, separating language-specific rules and task-specific rules amounted to re-designing an entirely new system for the new language. This experience suggests that a system that is designed to be as modular as possible, may be more easily adapted to new languages. As a modular system, cTAKES raises interest for adaptation to languages other than English. Initial experiments in Spanish for sentence boundary detection, part-of-speech tagging and chunking yielded promising results [30]. Some recent work combining machine translation and language-specific UMLS resources to use cTAKES for clinical concept extraction from German clinical narrative showed moderate performance [80]. More generally, the use of word clusters as features for machine learning has been proven robust for a number of languages across families [81].

Similarly to work in English, the methods for Named Entity Recognition (NER) and Information Extraction for other languages are rule-based [82, 83], statistical, or a combination of both [84]. With access to large datasets, studies using unsupervised learning methods can be performed irrespective of language, as in Moen et al. [85] where such methods were applied for information retrieval of care episodes in Finnish clinical text. Knowledge-based methods can be applied when terminologies are available, e.g. extending information contained in structured data fields with information from Danish clinical free-text with dictionary-based approaches for the study of disease correlations [86] or adverse events [87]. For German, extracting information from clinical narratives for cohort building using simple rules was successful [88].

NER essentially focuses on two types of entities: personal health identifiers in the context of clinical document de-identification [56, 57, 79, 79, 89–92] and clinical entities such as diseases, signs/symptoms [93], procedures or medications [61, 94–100], as well as their context of occurrence: negation [101], assertions [102, 103] and experiencer (i.e. whether the entities are relevant to the patient or a third party such as a family member or organ donor).

Systems addressing a task such as negation may be easily adapted between languages of the same family that express negation using similar syntactic structures as is the case for English and Swedish [104, 105], English and German [106], English and Spanish [107, 108], or even English, French, German and Swedish [109]. However, it can be difficult to pinpoint the reason for differences in success for similar approaches in seemingly close languages such as English and Dutch [110].

Another important contextual property of clinical text is temporality. Heideltime is a rule-based system developed for multiple languages to extract time expressions [111]. It has been adapted for clinical text in French [112] and Swedish [113].

Global concept extraction systems for languages other than English are currently still in the making (e.g. for Dutch [114], German [115] or French [116, 117]).

The entities extracted can then be used for inferring information at the sentence level [118] or record level, such as smoking status [119], thromboembolic disease status [7], thromboembolic risk [120], patient acuity [121], diabetes status [100], and cardiovascular risk [122].

#### Applications

There are a number of studies describing applications relying on some NLP preprocessing. Jacobson et al. [123] use deep learning to detect healthcare associated infections in Swedish patient records. Lopprich *et al* [124] describe a system using NLP methods for German to classify the diagnoses of Multiple Myeloma patients at Heidelberg University Hospital. The high average F1-scores demonstrate the suitability of the investigated methods. However, it was also shown that there is no best practice for an automatic classification of data elements from free-text diagnostic reports. A study on Georgian medical records, where documents were classified into types (Ultrasonography, X-ray and Endoscopy) and clinical categories (e.g. Thyroid, Biliary system) showed promising results, and highlights early work in an understudied, highly agglutinative language [125].

Metzger et al. [126] show how the development of machine learning-based classifiers using free-text data can be used to identify suicide attempts in a French Emergency Department with promising results (70.4-95.3% F1), demonstrating that the quality of epidemiological indicators can be improved by these types of approaches as opposed to manually coded information. Grouin et al. [120] show that information extraction from clinical records can sucessfully be used to automatically compute a cardio-vascular alert score on par with experts. Similarly, Takano et al. [127] use NLP to analyze Japanese patients cue-recalled memories to automatically determine memory specificity, an important indicator in the diagnosis of memory dysfunctions. NLP-based systems have been integrated into a clinical workflow for assisting clinical decision making or contributing to the construction of large health information system such as data warehouses. For instance, the Bulgarian system *BITool* is used for the construction of the register of diabetic patients in Bulgaria, which contains over 100 million de-identified reimbursement requests from all general practitioners and specialists in the country for a 3 year period [100].

### Discussion

As we enter an era where big data is pervasive and EHRs are adopted in many countries, there is an opportunity for clinical NLP to thrive beyond English, serving a global role.

#### How to develop a clinical NLP application in a language other than English?

Research on the use of NLP for targeted information extraction from, and document classification of, EHR text shows that some degree of success can be achieved with basic text processing techniques. It can be argued that a very shallow method such as lexicon matching/regular expressions to a customized lexicon/terminology is sufficient for some applications [128]. For tasks where a clean separation of the language-dependent features is possible, porting systems from English to structurally close languages can be fairly straightforward. On the other hand, for more complex tasks that rely on a deeper linguistic analysis of text, adaptation is more difficult.

In summary, the level of difficulty to build a clinical NLP application depends on various factors including the difficulty of the task itself and constraints linked to software design. Legacy systems can be difficult to adapt if they were not originally designed with a multi-language purpose.

#### Where are the best opportunities?

Clinical NLP in any language relies on methods and resources available for general NLP in that language, as well as resources that are specific to the biomedical or clinical domain.

In this respect, English is by far the most resource-rich language, with advanced tools dedicated to the biomedical domain such as part-of-speech taggers (e.g. MedPOST [129]), parsers (e.g. GATE [130], Charniak-McClosky [131], enju [132]), biomedical concept extractors (e.g. MetaMap [133], cTAKES [134, 135], NCBO [136]). For other languages, data and resources are sometimes scarce.

The UMLS (Unified Medical Language System [137]) aggregates more than 100 biomedical terminologies and ontologies. In its 2016AA release, the UMLS Metathesaurus comprises 9.1 million terms in English followed by 1.3 million terms in Spanish. For all other languages, such as Japanese, Dutch or French, the number of terms amounts to less than 5% of what is available for English. Additional resources may be available for these languages outside the UMLS distribution. Details on terminology resources for some European languages were presented at the CLEF-ER evaluation lab in 2013 [138] for Dutch [139], French [140] and German [141].

Medical ethics, translated into privacy rules and regulations, restrict the access to and sharing of clinical corpora. Some datasets of biomedical documents annotated with entities of clinical interest may be useful for clinical NLP [59]. However, there are currently no sharable clinical datasets comparable to the i2b2 datasets [142, 143], the ShARe corpus [144], the THYME corpus [145, 146] or the MIMIC corpus [147] in languages other than English except the Turku Clinical TreeBank and PropBank [32, 38, 148] in Finnish and the small subset of 100 patient pseudonymized records in the Stockholm EPR PHI Pseudo Corpus [92] in Swedish, and the examinations clinical texts of the MedNLPDoc corpus in Japanese [149], albeit only with document-level annotation.

Past experience with shared tasks in English has shown international community efforts were a useful and efficient channel to benchmark and improve the state-of-the-art [150]. The NTCIR-11 MedNLP-2 [151] and NTCIR-12 MedNLPDoc [149] tasks focused on information extraction from Japanese clinical narratives to extract disease names and assign ICD10 codes to a given medical record. The CLEF-ER 2013 evaluation lab [138] was the first multi-lingual forum to offer a shared task across languages. It resulted in a small multi-lingual manually-validated reference dataset [70] and prompted the development of a large gold-standard annotated corpus of clinical entities for French [59], currently in use in a clinical named entity recognition and normalization task in the CLEF eHealth evaluation lab [152, 153]. Our hope is that this effort will be the first in a series of clinical NLP shared tasks involving languages other than English. The establishment of the health NLP Center as a data repository for health-related language resources (www.center.healthnlp.org) will enable such efforts.

In summary, there is a sharp difference in the availability of language resources for English on one hand, and other languages on the other hand. Corpus and terminology development are a key area of research for languages other than English as these resources are crucial to make headway in clinical NLP.

#### How do we best leverage existing data and tasks?

**Leveraging resources for English.** The resource availability for English has prompted the use of machine translation as a way to address resource sparsity in other languages. Off-the-shelf automatic translators, e.g. Google translate, were found to have the potential to reduce language bias in the preparation of randomized clinical trials reports language pairs [154]. However, it was shown to be of little help to render medical record content more comprehensible to patients [155]. A systematic evaluation of machine translation tools showed that off-the-shelf tools were outperformed by customized systems [156]; however, this was not confirmed when using a smaller in-domain corpus [157]. Encouragingly, medical speech translation was shown to be feasible in a real clinical setting, if the system focused on narrowly-defined patient-clinician interactions [158]. Further work focused on acquiring and evaluating targeted resources [54, 55, 159].

Machine translation is used for cross-lingual Information Retrieval to improve access to clinical data for non-native English speakers. Successful query translation (for a limited set of query terms) was achieved for French using a knowledge-based method [160]. Query translation relying on statistical machine translation was also shown to be useful for information retrieval through MEDLINE for queries in French, Spanish [161] or Arabic [162]. More recently, custom statistical machine translation of queries was shown to outperform off-the-shelf translation tools using queries in French, Czech and German on the CLEF eHealth 2013 dataset [163]. Interestingly, while the overall cross-lingual retrieval performance was satisfactory, the authors found that better query translation did not necessarily yield improved retrieval performance.

More recently, machine translation was also attempted to adapt and evaluate cTAKES concept extraction to German [80], with very moderate success. Making use of multilingual resources for analysing a specific language seems to be a more fruitful approach [152, 153, 164]. It also yielded improved performance for word sense disambiguation in English [165].

**Learning from other languages.**

The common clinical NLP research topics across languages prompt a reflexion on clinical NLP in a more global context.

Recent work on negation detection in English clinical text [166] suggests that the ability to successfully address a particular clinical NLP task on a particular corpus does not necessarily imply that the results can be generalized without significant adaptation effort. This may hold true for adaptations across languages as well, and suggests a direction for future work in the study of language-adaptive, domain-adaptive and task-adaptive methods for clinical NLP. The LORELEI [167] initiative aims to create NLP technologies for languages with low resources. While not specific to the clinical domain, this work may create useful resources for clinical NLP.

Interestingly, segmentation with lack of spacing in Japanese [33] could be successfully applied to English text where spacing between words was removed such as in Character Recognition (OCR) where word spacing is often not captured properly. Duque et al. [165] show that multilingual ressources can be useful for processing English text: for a word sense disambiguation task, multilingual resources yield a 7% improvement in performance, compared to monolingual resources.

## Conclusion

In summary, we find a steady interest in clinical NLP for a large spectrum of languages other than English that cover Indo-European languages such as French, Swedish or Dutch as well as Sino-Tibetan (Chinese), Semitic (Hebrew) or Altaic (Japanese, Korean) languages. Our review of recent studies shows that (1) the field is maturing, (2) researchers in the community have access to datasets, which enables them to develop powerful methods to address clinical NLP tasks of interest such as EHR de-identification, clinical entity recognition, normalization and contextualization. We identified the need for shared tasks and datasets enabling the comparison of approaches within- and across- languages. Furthermore, the challenges in systematically identifying relevant literature for a comprehensive survey of this field lead us to also encourage more structured publication guidelines that incorporate information about language and task. We suggest that efforts in analyzing the specificity of languages and tasks could contribute to methodological advances in adaptive methods for clinical NLP.

## Abbreviations

ACL: Association for computational linguistics
AMIA: American medical informatics association
CLEF: Cross language evaluation forum
cTAKES: clinical Text analysis and knowledge extraction system
EHR: Electronic health record
ICD10: International Classification of Diseases, 10th revision
IMIA: International medical informatics association
NLP: Natural language processing
SNOMED-CT: Systematized nomenclature of medicine - clinical terms
UMLS: Unified medical language system
WHO: World health organization
